# 芦可替尼治疗PCM1-JAK2融合基因阳性骨髓增殖性肿瘤伴嗜酸性粒细胞增多症一例

**DOI:** 10.3760/cma.j.issn.0253-2727.2021.08.015

**Published:** 2021-08

**Authors:** 颖 宋, 育榕 张, 叶 韩, 茵 王, 艳青 李, 晔 柴, 鹏云 曾, 玲玲 岳, 重阳 吴

**Affiliations:** 兰州大学第二医院血液科，兰州 730000 Department of Hematology, the Second Hospital of Lanzhou University, Lanzhou 730000, China

患者，男，47岁，因“耳鸣2年，加重伴乏力、头晕2个月”于当地医院就诊。查血常规：WBC 37.6×10^9^/L，中性粒细胞0.76％，淋巴细胞0.03％，单核细胞0.037％，HGB 74 g/L，PLT 72×10^9^/L，予以输血治疗，症状无缓解。于2020年12月27日就诊于我院，以“白血病”收住院。入院查体：全身皮肤黏膜无黄染、瘀点及瘀斑，睑结膜、口唇、甲床中度苍白，浅表淋巴结未触及肿大，无胸骨压痛；心肺查体无异常；肋缘下约2 cm可触及肿大肝脏，表面光滑，无触痛；脾大（甲乙线3 cm，甲丙线4 cm，丁戊线-3 cm），质硬；双下肢无水肿；血常规：WBC 36.78×10^9^/L，中性粒细胞0.73％，淋巴细胞0.04％，单核细胞0.03％，嗜酸性粒细胞0.21％（绝对计数7.59×10^9^/L），HGB 77 g/L，PLT 80×10^9^/L，网织红细胞1.1％；血液生化检查：LDH 252 U/L（参考值120～250 U/L），直接胆红素8.6 µmol/L（参考值0.1～6.8 µmol/L），白蛋白35.6 g/L（参考值40.0～55.0 g/L），其余未见异常。血β_2_微球蛋白3790 µg/L（参考值609～2366 µg/L）；铁蛋白554 µg/L（参考值30～400 µg/L），叶酸1.28 µg/L（参考值4.20～19.80 µg/L），维生素B12>2000 ng/L（参考值197～771 ng/L）。腹部彩超：肝肋缘下探及肝实质长约2.6 cm，形态饱满，被膜光整；脾脏脾门处厚6.5 cm，肋缘下4.0 cm，厚4.5 cm，形态饱满，内部回声均匀；胆、胰、双肾未见明显异常。骨髓象：增生明显活跃，粒系占91％，红系占4％，粒∶红＝22.75∶1；粒系比例增高，部分粒细胞胞质内可见增多增粗颗粒，嗜酸性粒细胞比例增高（9.5％）；红系比例减低，形态未见明显异常，淋巴细胞比例减低，巨核细胞减少（全片共见4个）。外周血涂片可见幼稚阶段粒细胞及有核红细胞（2个），泪滴样红细胞易见，嗜酸性粒细胞增多（14％）。骨髓免疫分型：成熟淋巴细胞比例减低，未见异常细胞；髓系原始细胞比例极低，未见异常表达；粒细胞比例正常，成熟阶段偏多；嗜酸性粒细胞比例升高（15.29％）。骨髓病理：造血组织增生明显活跃，脂肪组织增生减低；粒系增生，比值增高，幼稚前体细胞散在，中幼及以下阶段细胞为主，嗜酸性粒细胞散在可见，形态均未见明显异常；红系增生减低，巨核细胞偏少（0～3个/高倍视野），散在分布，分布不均，少数胞体小细胞；淋巴细胞散在，浆细胞散在或小簇分布；区域性纤维组织增生；网银染色0～1级。因基因检查结果回报较慢，患者出院等待结果，6 d内白细胞计数由11.33×10^9^/L升至69.2×10^9^/L，门诊行外周血涂片未发现原始细胞，仍以中晚幼粒细胞为主，分类100个有核细胞，见嗜酸性粒细胞12个，有核红细胞2个，可见泪滴样红细胞。予以羟基脲1.5 g/d，2 d后白细胞计数升至88.11×10^9^/L，遂将羟基脲加量至3 g/d，5 d后白细胞计数38.8×10^9^/L。染色体及基因相关检查回报：骨髓染色体核型46,XY[20]；高通量测序未发现异常；慢性髓性白血病相关基因BCR-ABL（p190型、p210型、p230型）阴性；骨髓增殖性肿瘤（MPN）常见基因突变JAK2V617F、JAK2 exon12、MPL-W515L/K、CALR均为阴性；原发嗜酸性粒细胞增多症相关基因PDGFRA、PDGFRB、FGFR1重排阴性，ETV6-FLT3、BCR-JAK2、ETV6-JAK2、ETV6-ABL1融合基因阴性，PCM1-JAK2融合基因阳性。参照2016年WHO髓系肿瘤和急性白血病分类，明确诊断为“PCM1-JAK2融合基因阳性的骨髓增殖性肿瘤伴嗜酸性粒细胞增多症（MPN-Eo）”。因PCM1-JAK2融合基因为阳性，给予JAK2抑制剂芦可替尼10 mg/d治疗，7 d后白细胞计数降至正常，腹胀较前减轻，因患者贫血症状明显，遂输注红细胞1000 ml，继续芦可替尼10 mg/d治疗3 d，减至5 mg/d维持至今。

